# Moving enhanced recovery after surgery from implementation to sustainability across a health system: a qualitative assessment of leadership perspectives

**DOI:** 10.1186/s12913-020-05227-0

**Published:** 2020-04-26

**Authors:** Leah Gramlich, Gregg Nelson, Alison Nelson, Laura Lagendyk, Loreen E. Gilmour, Tracy Wasylak

**Affiliations:** 1 University of Alberta, 214 CSC Royal Alexandra Hospital, Edmonton, Alberta Canada; 2 University of Calgary, Foothills Medical Center, Calgary, Alberta Canada; 3 Alberta Health Services, Calgary, Alberta Canada

**Keywords:** Sustainability, Implementation, Leadership perspectives, Enhanced recovery after surgery

## Abstract

**Background:**

Knowledge Translation evidence from health care practitioners and administrators implementing Enhanced Recovery After Surgery (ERAS) care has allowed for the spread and scale of the health care innovation. There is a need to identify at a health system level, what it takes from a leadership perspective to move from implementation to sustainability over time. The purpose of this research was to systematically synthesize feedback from health care leaders to inform further spread, scale and sustainability of ERAS care across a health system.

**Methods:**

Alberta Health Services (AHS) is the largest Canadian health system with approximately 280,000 surgeries annually at more than 50 surgical sites. In 2013 to 2014, AHS used a structured approach to successfully implement ERAS colorectal guidelines at six sites. Between 2016 and 2018, three of the six sites expanded ERAS to other surgical areas (gynecologic oncology, hepatectomy, pancreatectomy/Whipple’s, and cystectomy). This research was designed to explore and learn from the experiences of health care leaders involved in the AHS ERAS implementation expansion (eg. surgical care unit, hospital site or provincial program) and build on the model for knowledge mobilization develop during implementation. Following informed consent, leaders were interviewed using a structured interview guide. Data were recorded, coded and analyzed qualitatively through a combination of theory-driven immersion and crystallization, and template coding using NVivo 12.

**Results:**

Forty-four individuals (13 physician leaders, 19 leading clinicians and hospital administrators, and 11 provincial leaders) were interviewed. Themes were identified related to Supportive Environments including resources, data, leadership; Champion and Nurse coordinator role; and Capacity Building through change management, education, and teams. The perception and role of leaders changed through initiation and implementation, spread, and sustainability. Barriers and enablers were thematically aligned relative to outcome assessment, consistency of implementation, ERAS care compliance, and the implementation of multiple guidelines.

**Conclusions:**

Health care leaders have unique perspectives and approaches to support spread, scale and sustainability of ERAS that are different from site based ERAS teams. These findings inform us what leaders need to do or need to do differently to support implementation and to foster spread, scale and sustainability of ERAS.

## Background

Enhanced Recovery After Surgery (ERAS) is an approach that applies evidence-based care to pre-operative, intra-operative and post-operative aspects of a patient’s surgical journey, building upon audit of practice and strategies to support change management. Reports of ERAS care predominantly describe implementation within a particular surgical area (eg. colorectal, [1, 2] gynecologic oncology [3]) and its impact on outcomes such as safety, cost, efficiency and effectiveness [4].

Qualitative research and evaluation studies have identified a variety of barriers and facilitators that influence ERAS care implementation, largely through interviews with ERAS nurse coordinators or champions (i.e., healthcare providers heavily involved in ERAS) [5–7]. In these studies, the use of a bottom-up approach and knowledge transfer through peer influence was consistently identified as important. In addition, sustainability planning was felt to be a promising strategy for normalization of ERAS [7].

Although ERAS implementation has been studied, there is less information available about spread, scale and sustainability of this healthcare innovation. Spread of innovation has been defined as horizontal diffusion of best practices within an organization [8]. It is influenced by local actions and context and relies on local (site or unit based) healthcare providers, managers and leaders. Local actions and influences are leveraged by health system leaders to promote spread of evidence based best practice. Scale has been defined as the use of more vertical strategies along the spectrum from patients and families, to front line staff, unit managers, department managers and directors to executive leadership within the healthcare system [8, 9]. Scale implies altered resources and support along with formal authority to apply common factors across multiple environments with a single approach [8]. The Institute for Healthcare Improvement (IHI) has published a white paper that healthcare organizations can use to sustain improvements in the safety, efficiency and effectiveness of patient care [10]. This framework highlights that the key to sustaining improvements, such are seen with ERAS implementation, is to focus on the daily work of frontline managers with support of a high-performing management system. Leadership at senior levels (board of directors, vice presidents, senior operating officers, chairs and chiefs of divisions) is necessary to cultivate and lead improvement throughout the organization, but smaller incremental steps within service delivery units can also set the stage for whole system change.

While there is evidence demonstrating the impact of ERAS implementation [2, 4] on outcome, there is a need to better understand what enables spread, scale and sustainability of ERAS across a health system. ERAS clinical and administrative leaders at all levels of the organization have insight into and accountability for quality surgical care as well as influence. The purpose of this qualitative research was to systematically seek feedback from health care leaders to inform spread, scale and sustainability of ERAS across the health system.

### Context

In Alberta, Canada, ERAS was initially implemented and studied [2] in colorectal surgery in Alberta Health Services (AHS). AHS is North America’s largest integrated publicly funded health system, serving over 4 million people with over 280,000 surgeries per year in over 50 surgical sites. The ERAS innovation was subsequently spread beyond colorectal surgery to other surgical areas in AHS, in a planned implementation informed by research based on the QUERI model and Theoretic Domains Frameworks (TDF) [11]. In this work [12], data from patients and healthcare professionals was analyzed for barriers or enablers, organized into a framework that included individual to organization impact, and areas of focus for guideline implementation. Barriers and enablers were thematically aligned in order to allow better insight into implementation issues related to sustainability through application of QUERI considerations articulated by Stetler et al. [13] The data identified Capacity Building (through teams, tools, communication, and education); Clinical Care Elements (in specific areas including nutrition, mobilization, hydration, pain and symptom control, best surgical practices); and Supportive Environment (including data-related issues, supporting patients, and human resource availability such as ERAS nurse coordinators) as key areas for focus. Supporting patients was also identified as an important theme by both patients and health care providers [14]. Use of TDF provided a model for exploring barriers, enablers, and strategies to effect practice change. Through the use of a formal program (ERAS and the ERAS implementation Program), phasing in implementation, and using an integrated approach to mobilizing adoption of evidence-based practice, processes, tools and strategies were spread from early adopter sites to include the majority of centers performing colorectal surgery in AHS. Building upon theory based implementation has aided spread and scale of ERAS across multiple surgical areas and unique surgical sites.

Given the integrated nature of the Alberta health system, understanding system level considerations for successful implementation, spread, scale and sustainability was defined a priori as a goal of the ERAS Alberta research plan that was supported through the Strategic Clinical Networks™ (SCNs – the engines of innovation in the health system) and a Partnership for Research and Innovation in the Health System (PRIHS) grant.

In 2015–2017 three of the six sites expanded ERAS to other surgical areas within the site (see Table 1). The continued progress and expansion of ERAS provided opportunity for health care leaders involved at multiple levels of the healthcare system (i.e., surgical unit, hospital site or provincial operations) to gain ERAS experience.

**Table 1:** AHS Hospital Site Implementation of ERAS Protocols

Site	Colorectal	Gynecological Oncology	Pancreas	Liver
A	Fall 2013			
B	Fall 2013			
C	Fall 2014			
D	Fall 2014	Fall 2016	Winter 2016	Summer 2017
E	Summer 2014		Winter 2016	Summer 2017
F	Summer 2014	Winter 2017		

## Methods

### Research design, data collection

AHS health care leaders involved in ERAS at site (local hospital) or program (provincial) levels were identified using purposive sampling [14], contacted by email and invited to participate in a short interview (Additional file 1). Interviewees were categorized by location of work site (i.e., hospital, zone or provincial) and role (physician (i.e., surgeon or anaesthetist) clinician (nursing, nurse coordinators, dietetics, pharmacy, physiotherapy, including nurse coordinators) or provincial (leaders with a cross-province perspective because of their ERAS or leadership role). Sixty–nine individuals were invited to participate and forty-three interviews were conducted (44 interviewees; one provincial interview had two participants). (See Table 2).

**Table 2:** Categorization of Interviews by Site and Role

Site	Physician Leaders	Leading Clinicians & Hospital Administrators	Provincial Leaders	Total
**A**	2	4		**6**
**B**	1	2		**3**
**C**	1	2		**3**
**D**	3	4		**7**
**E**	3	4		**7**
**F**	3	3		**6**
**Provincial**			11	**11**
**Total**	**13**	**19**	**11**	**43**

Individual interviews were conducted following informed consent by telephone by a member of the research team (LGi) experienced in qualitative interviewing. Semi-structured interviews were conducted using an interview guide (Additional file 1), audio recorded and transcribed. Interviewing was conducted from October – December 2017. The interviewer (LGi) was familiar with the ERAS context and performed the preliminary analysis. An analyst experienced in qualitative research (LL) but unfamiliar with the data or context did subsequent analysis of the interview data, providing a balance of perspectives in analysis and themes generation. This work adheres to Standards for Reporting Qualitative Research (SRQR).

This study was funded through a PRIHS grant from Alberta Innovates. This study was reviewed and approved by the Conjoint Health Research Ethics Board of the University of Calgary, Alberta, Canada (ID: REB14–1506 MOD1). Participants provided verbal consent for participation. This study has been presented in abstract at the European Society of Nutrition and Metabilism in 2019 [15].

### Data analysis

Transcribed interviews were assigned a unique identifier with removal of individual names. Transcripts were imported into NVivo 11 and coded by question and response with minimal additional coding for topics. The interviewer (LGi) reviewed the coding and generated a list of preliminary themes, with review and input by LG. Subsequent qualitative analysis was a combination of theory-driven immersion and crystallization [16] and template coding [17, 18] using NVivo 12 Plus and conducted by LL. The research team met regularly to review and revise analysis.

#### Generation of topics and themes

Interviews were read in depth, and an initial list of topics were defined and search terms generated. Topics were considered for inclusion in this report if they were prominent; that is, if there was high frequency (i.e., frequent references to the topic), wide breadth (i.e., mentioned by multiple interviewees) or both. Prominent topics were further explored by reading in depth for context and refined until saturation was reached. These topics were initially mapped onto the implementation themes identified in previous Knowledge Translation work (i.e., Capacity Building, Clinical, Supportive Environment, Culture) [12] . Further refinement yielded four themes (Supportive Environment, ERAS nurse coordinators and ERAS Champions, Capacity Building, Barriers and Enablers). The themes include 12 prominent topics that cut across most interviews, professions and site or provincial perspectives. Topics related to “clinical themes” previously identified [12] were not prominent in leadership perspectives.

#### Stages of ERAS: initiation and implementation, spread and scale, sustain

Three stages of ERAS (i.e. Initiation and Implementation, Spread and Scale, and Sustain) are considered, derived from the interview discussion context.

The “initiation and implementation” stage includes interviewee references to ERAS launching and getting started or implementing and maintaining ERAS. The “spread and scale” stage includes interviewee references to extending ERAS beyond the initial surgical area to all surgical areas within a site, or spread across sites. The “sustain” stage includes interviewee discussion about what is needed to expand and sustain ERAS (defined as ERAS becomes standard of practice within AHS) at the provincial level across all facilities providing surgical care. It acknowledges the need for tailoring the ERAS intervention to unique sites and program areas. It is recognized that these stages may not be linear, but rather are iterative with back and forth between stages as new guidelines and sites are initiated.

## Results

This section reports the emergent themes and prominent topics of interviewee discussion. The themes are discussed by topic, across the stages of ERAS development. A selection of quotes that illustrate the topics are included in a table for each theme. Additional quotes that represent a range of viewpoints and the stages of ERAS development are included in Additional file 2 to illustrate the breadth of interviewee discussion. Quotes that reference more than one topic are often selected to efficiently illustrate the data. Quotes are identified by role only, to maintain confidentiality.

The emergent themes and prominent topics of interviewee discussion have been mapped across the stages of ERAS through the implementation to sustainment process. Key activity features required of leadership are aggregated and highlighted in Table 3, Key Findings. Themes identified include supportive environment, role of nurse coordinators and champions and the need for building capacity. These themes are constant throughout the implementation to sustainment process but they evolve. At launch of ERAS in a unique area, visible support, funding of nurse coordinators and endorsement is vital. Leadership also has a role to play regarding identifying and promoting support for processes such as communication, compliance measures and data. With spread and scale of ERAS, leaders must demonstrate and set expectations that Enhanced Recovery will expand and plan for sufficient resources. Leaders must plan for standards, for data and communication and continue to build capacity across surgical areas and sites. In order to plan for and to succeed at sustainability, leaders must balance resources used for ERAS with benefits, reinforce practice changes, identify competing change initiatives, identify consistent expectations around ERAS as standard of care, and establishment of core data sets and a plan for audit of practice. Based on the interviews, the role of leaders evolves during the implementation to sustainment process.

**Table 3:** **Key Findings**

	Stages of ERAS Implementation/ Sustainability		
	Initiate & Implement	Spread & Scale	Sustain
**Themes**			
**Supportive Environment**	**What provides a supportive environment?**		
**Leadership**	Strong, visible support at every level of leadership facilitates early stages	Leaders setting expectations that ERAS will continue and expand	Leaders’ pride in outcomes and confidence that ERAS practices will be standard AHS practice
**Resources**	Funding for tools and practice changes	Plan for sufficient resources for expansion	Explicit resources sustainability plan
**Data**	Essential for engagement of staff and physicians in practice change	Establish data standards; provide resources for data management	Establish core dataset; align with AHS data collection; assess skillset required for data management
**Nurse coordinators & Champions**	**How do these roles add value?**		
**Nurse coordinators**	Build enthusiasm	Encourage compliance	Maintain practices
**Champions**	Engage peers	Address misperceptions; migrate ERAS	Practice ERAS as usual standard of care
**Building Capacity**	**What builds ERAS capacity?**		
**Change Management**	Include change management plan from outset	Encourage ERAS spread through peers	Changes need to be reinforced as standard practice
**Education**	Essential for staff engaging in new practices	Support transfer of “lessons learned” across change areas	Ongoing need for continuous education (e.g., as staff changes)
**Teams**	Regular ERAS team meetings within change area beneficial	Support relationship development across change areas	Collaboration at provincial level supports ERAS practice
**Enablers**	**What enables ERAS development?**		
**Multiple Guidelines**	Some benefits when staff have positive ERAS experience	Ensure ERAS resources match scope of spread to multiple guidelines	Assess volume of change initiatives affecting staff
**Consistency**	Consistency of ERAS processes supports early stages	Consistency across patients spread and scale	Consistent expectations and supporting resources to enable culture change
**Compliance**	Leadership expectations for compliance support ERAS initiation and implementation	Support the use of ERAS data to encourage compliance	Establish minimum AHS standards (based on international standards)
**Outcomes**	Communicate initial ERAS outcomes frequently to engage staff in change	Use outcome data to support areas where positive changes are slipping	Outcome data supports positive reinforcement of good outcomes for staff and patients

To focus the findings and to integrate system level perspectives for practical application and building upon TDF (eg. “who needs to do what differently” at the system level) each theme has been assigned a key question. (eg. “What provides a supportive environment?”). These themes are further developed in the following sections.

### Supportive Environment Theme: What provides a supportive environment for ERAS development? (Table 4).

The main topics discussed were: the role of leadership, having sufficient resources, and the importance of data.

**Table 4:** Supportive Environment Theme; Quotes by Topic

Supportive Environment Theme	What provides a supportive environment for ERAS development?
*Topic*	*Quote*
*Leadership*	*We had very strong physician leadership from the beginning, both with general surgery, the colorectal guys, as well as with anesthesia. The fact that they bought in right from the beginning and were visible champions, I think that made a huge difference in terms of being able to move ERAS forward. Once that commitment was seen I think we saw the nursing leadership, the operational leadership also make that commitment. And so, I think that’s really key in this work.* Clinician_11
*Resources*	*From the system wide, running the program thing…I think that if we are going to implement this across the entire hospital, which is eventually where I think we need to go, you know vascular, urology, gynecology, or orthopedics. There is going to be some more financial support for nursing management, for the enhanced recovery after surgery coordinator nurse position. Not only for management program, but also for data analysis work that needs to happen. So you know more funding for that.* Physician_4
*Data*	*So, it’s kind of tricky that way. I would like to see it expand to all the surgeries and have ERAS for all because it does make a difference. I’m worried about sustainability because ERAS for colorectal has been at this site for quite some time. But research has shown that if the data isn’t monitored, it does drop off and it’s so true. They’re just not self-sustained. They need somebody to still have the meetings, still bring the group together, and still talk about the data otherwise I’m fearful that it will just drop off.* Clinician_15

### Leadership

Interviewees discussed the importance of leadership within all themes and at all stages of ERAS development. Examples of the roles of leadership during initiation and implementation included building understanding of ERAS and providing strong and visible support of ERAS to set the tone for the practice changes ERAS requires. Leadership roles were essential to ensure adequate staffing and resources for ERAS. Interviewees sometimes linked the role of leaders to site culture, suggesting the role of leaders included exercising authority to institute ERAS changes at appropriate levels (site, zone or AHS wide). The role of leaders in sustaining ERAS was to reinforce positive outcomes attributed to ERAS, and to make decisions that indicated ongoing, consistent support of ERAS becoming standard AHS practice.

### Resources

The importance of having sufficient resources made available to the surgical area working with ERAS as a key part of a supportive environment was articulated. Leaders were instrumental in resource allocation. The most common examples of resource needs were: funding for the nurse coordinator position and ERAS data management. Interviewees were consistent in their beliefs that ERAS practices benefited patients and providers, and in their desire to expand and sustain ERAS within AHS; yet, they were also concerned about the resources required to do so. Many suggested reviewing current ERAS data management practices and establishing standards as a way of managing costs. Others suggested that continued spread of ERAS would provide cost savings and allow resource efficiencies. 

### Data

The benefits of having ERAS data and the challenges of its management were topics raised by almost all. Data demonstrating ERAS practice changes and improved outcomes was a commonly cited motivating factor at all leadership levels. Frequently mentioned challenges were inconsistent, resource intensive data collection processes and an overwhelming number of reported measures. Spreading ERAS to new surgical areas multiplied the problem. Common suggestions for sustainment related to standards for data definitions, minimal data set, data collection practices and alignment with other AHS data practices.

### Nurse coordinators and Champions Theme: How do these roles add value? (Table 5).

With each ERAS implementation, experienced nurse coordinators were hired to support and promote ERAS, and site based surgeon, anesthesia, and clinical operations champions were identified. The roles of ERAS nurse coordinators and ERAS champions were frequently mentioned as essential elements of ERAS across stages.

**Table 5:** Nurse Coordinators and Champions Theme; Quotes by Topic

Nurse coordinators and Champions Theme	How do these roles add value?
*Topic*	*Quote*
*ERAS Nurse Coordinators*	*From what is working, I mean definitely having a nurse coordinator at each site, that’s working. And I see that is very important, especially up front to getting it launched, having the connections on site…. So having the onsite at the beginning of the implementation is definitely working* Provincial_08
*ERAS Champions*	*It’s absolutely vital that there’s champion and both formal leadership champions and informal champions and leaders for each of the professional groups, for each of the clinical service areas... And every place along the pathway that there’s patient care delivered, patient service delivered, there has to be champions around making things better for patients and providers. And if the focus is not on both of those, then it doesn’t work.* Provincial_13

### Nurse coordinators

The acknowledgement of the importance of ERAS nurse coordinators and secure funding for them was ubiquitous throughout interviews. The coordinators built enthusiasm for change, encouraged compliance and maintained ERAS practices over the longer term. Successful nurse coordinator characteristics included strong change management skills, respectful understanding of the surgical area, building good relationships, and being passionate about ERAS. They were often viewed as the face of ERAS. Interviewees frequently stated that ERAS practices declined in the absence of coordinator presence. The challenges raised about the nurse coordinator role centred around workload, staff turnover, and concerns that the current levels of nurse coordinator support were not sustainable, especially in discussion of ERAS expansion.

### Champions

The ERAS champion role was seen as essential to all stages. Champions were viewed as physicians and operational leaders who understood and visibly supported ERAS formally or informally. Effective ERAS champions were seen as professional peers who were vocal, respected, and had sufficient confidence to help the team weather the inevitable challenges of change. ERAS champions corrected misperceptions or presented a balancing view that countered stories of negative experiences attributed to ERAS. Peer champions, across and within disciplines, that worked in different surgical areas were especially effective in spreading and building a culture of ERAS as the usual standard of care.

### Capacity Building: What builds ERAS capacity? (Table 6).

Topics identified that helped build ERAS capacity include: change management, education and the need to support teams.

**Table 6:** Capacity Building Theme; Quotes by Topic

Capacity Building Theme	What builds ERAS capacity?
*Topic*	*Quote*
*Change Management*	*In terms of challenges, the biggest thing was just the constant need for reinforcement about what we were doing, both on the surgeon side and the nursing side. That takes, once you implement a change…it seems to take a couple of years for them to adopt it and use it on a daily routine basis. Just ‘cause you’re so used to doing things the old ways. So I think the challenge is that people tend to fall backwards and the solution to that is just constantly reinforcing the value…* Physician_6
*Education*	*The thinking about mobilization and nutritional supplements and the modern fasting guidelines, we are not just initiating that education and teaching with the colorectal patients. We talk about that with all of our patients that are coming through the pre-admission clinic for all of our services. So, I think that there are a lot of aspects of ERAS that have sort of migrated over. The mobilization, the intraoperative fluid, I think we’re seeing more and more of that just become the change in how we deliver care. So I don’t think it’s a big leap to go add another service on full bore. That will be really easy because, like the nursing personnel, ERAS way of managing patients is now become the way they do care…. I think most units and areas embrace this because it is better for the patient.* Clinician_8
*Teams*	*I think we worked as a team. We had regular meetings for feedback. And if the ERAS nurse had a question and we usually addressed it quickly. At least we tried to. We would brainstorm for things that hadn’t happened yet… Sometimes different physicians they are used to doing certain things in a certain way so they might not implement as early as others. People who kind of … people who jumped on the ERAS band wagon. Some people are more resistant to change than others. But overall it was slowly but surely… Everyone helped each other. Everyone has their own silo but overall people have been helpful. But there is an ERAS nurse that helps to put it all together.* Physician_12

### Change management

Interviewees stressed the importance of leadership building a sense of shared purpose to improve patient care through ERAS by bringing AHS staff and physicians together at ERAS initiation. Equipping ERAS nurse coordinators with formal change management training was suggested to increase change effectiveness. To spread and sustain ERAS, interviewees described using multiple education and communication formats with a variety of content that appealed to different ERAS groups. The importance of ongoing positive reinforcement to address change fatigue and pockets of change resistance was stressed.

### Education

Interviewees described the pivotal role of the nurse coordinator by providing ERAS education to frontline physicians, staff, and operational leaders. Education (often linked to patients and patient care) increased awareness and their adoption, providing the impetus for change. High quality and consistent ERAS patient education resources was valued as was the need to educate staff and physicians about the benefits of ERAS practices on patient outcomes. Provincial ERAS standards, education materials and templates for communication were viewed as resources that could facilitate ERAS spread, scale and sustainability.

### Teams

The importance of teams in building capacity for ERAS was evident at all stages. Physician leadership was viewed as instrumental in engaging all staff in ERAS; where physician support was mixed or lacking, interviewees reported challenges. Frequent, regular cross-professional meetings of ERAS teams were described as highly effective in building shared understanding of ERAS practices, relationship development, and as a way to encourage enthusiasm and overcome resistance to change. In addition, ERAS practices promoted within one team built capacity in other teams. Learning collaboratives promoted spread and scale of ERAS knowledge across sites, providing a forum for established teams to support capacity in new teams. The necessity of maintaining a strong sense of multi-professional teams to support ongoing reinforcement of ERAS practices was described.

### Barriers and Enablers: What hinders or enables ERAS development? (Table 7)

Four topics that figured prominently were: the discussion of outcomes, the effect of multiple guidelines being implemented within a site, the importance of consistency; and, the need for compliance.

**Table 7:** Barriers and Enablers Theme; Quotes by Topic

Theme	What hinders or enables ERAS development?
*Topic*	*Quote*
*Outcomes*	*So I think that showing people the data that good practice leads to good outcomes was a very powerful tool in- that’s probably the most convincing thing I could do…I guess it would be a short term laboring, that’s a management problem - where you do the change and you measure outcomes you get a short term view. So I think that was very good for that. I think that was the most powerful tool that I had. Again, the coordinator was very supportive of that.* Physician_8
*Multiple Guidelines*	*[Implementing one pathway…] It seemed to be very doable. When we got to multiple pathways, it wasn’t that it wasn’t doable, but what I started to hear from the sites…was more of a little bit of a sense of exhaustion and frustration with multiple things coming at once. So I started to hear “Okay I can handle one pathway” and especially from the site sponsors right? Through the site administrators, they are kind of like “yeah I can tap my one physician on the shoulder” around this one pathway. But when there is multiple pathways I’ve started to get calls from them or hear from them more about general frustration with provincial teams, with the networks. And I think it’s not because they don’t know it was coming but because it felt, all the sudden it felt a little bit overwhelming. Because we were recruiting people at the same time and then we were rolling out and that requires a larger role for some of the administrators.* Provincial_04
*Consistency*	*ERAS should become best practice for all patients. We need these standardized order sets for all patients - have to be out of bed day 0, all patients should have Ensure,* etcetera*. Then there would be a better uptake. But because we only have say 25–50% of patients are ERAS on units, I am speaking to post op, they’re the patients that are the most work. When really they are the patients that are getting the best care, if these measures are being done…. I don’t like to say ERAS anymore as I say enhanced recovery, because I feel like ERAS has gotten this negative definition to it where when you say enhanced recovery you can’t argue with that, right?* Clinician_16
*Compliance*	*Some it is organic, because everyone is getting off ward with some of the basic elements of ERAS. Some of the fine tuning stuff that are harder, like the big changes I think have been done, but some of the fine tuning in improving your compliance, no really active process on that. It’s challenging and truthfully I don’t how all of that is being achieved right now without having the learning collaborative and some of the provincial dedicated support really hands on. I think that the teams are doing as well as they can but I am sure it’s a challenge with the capacity and the ability of the coordinators to really be as involved because what we see is, unfortunately, without really good dedicated coordinator time hands-on on the unit working with the nurses, working with the physicians, advocating for change constantly, it’s really hard.* Provincial_06

### Outcomes

Relevance and importance of outcome was raised in all stages of ERAS development, often linked with the topics of resources, data, change management and compliance. In the initiation and implementation stages of ERAS development, a focus on patient outcomes was highly motivational to teams. Leaders spoke about the importance of champions and nurse coordinators having baseline data, initiating the ERAS changes, and then using short term outcomes to generate enthusiasm. Continued emphasis on patient, provider and healthcare system outcomes was suggested as a powerful way to positively reinforce and sustain ERAS practice, provided it could be done without substantially increasing workload.

### Multiple guidelines

Interviewees presented mixed views on the impact of initiation and implementation of additional ERAS programs after colorectal. Although some noted ease of implementation due to staff experience and practice spread, others suggested the variation of context, staff and surgical area practices meant implementation in a new area could still present challenges. Interviewees reported the most challenges when expansion to new surgical areas was not accompanied by increased resources. Some noted considerable effort was required to sustain ERAS in the original areas. Similar challenges were seen when competing priorities emerged (e.g., introduction of other quality improvement initiatives). Interviewees raised awareness of the need to account for provincial context when scaling ERAS. Some anticipated these tensions would ease when ERAS practices became the norm.

### Consistency

The desire for consistency across ERAS processes and supports, and across patient groups was identified. Lack of consistency in ERAS infrastructure (e.g., templates, documents, data definitions, collection or reporting methods) at initial implementation was viewed as a gap. Interviewees who perceived ERAS as best practice preferred to extend ERAS consistently across surgery groups, stating that that making ERAS practices (such as modern fasting, nutrition, hydration and mobilization guidelines) standard for all surgeries would ease implementation and benefit patients, staff and physicians. One of the commonly reported barriers to spreading ERAS was the tension between insisting on standardized ERAS processes vs allowing surgical areas to customize ERAS to their context.

### Compliance

One of the key outcome measure of ERAS implementation is compliance to the evidence based guidelines. It was perceived that where leaders set expectations for ERAS practices and where compliance to ERAS evidence based practices was measured through audit, the ERAS way would become the standard of care. Interviewees repeatedly stated that in absence of continued measurement of compliance, ERAS practices declined. There was some suggestion that optimal compliance measures for AHS were still being determined. Interviewees called for continued strong leadership at multiple levels in the organization (site, zones, AHS executives) to set the expectations of compliance with a subset of ERAS practices and aligned data as standard AHS practice.

## Discussion

Promoting health system transformation by spreading and scaling innovation requires dedicated action from health system leaders working locally and at the level of the health system [8]. With ERAS Alberta we illustrated common factors and created capacity for scale through common tools and resources and effected a culture shift. Proven interventions such as ERAS are potentially more amenable to scale through both bottom up and top down approaches necessary to foster practice change. Health system leaders can build upon spread and scale of innovation through promotion and adoption of ERAS and through cultivation of shared values, culture change in healthcare and relationship building on a foundation of trust and authenticity [19–21]. Leaders need to be kept informed of progress and measures of practice change and impact on outcome to help them continue to lead by setting expectations for both site and system performance.

Our previous research on ERAS implementation emphasized the importance of ensuring that healthcare professionals know what they need to do clinically and have the capability, opportunity and motivation to do this. The ERAS nurse coordinators have become Implementation and Quality Practice Change Specialists in surgery. They coordinate standard work and improvement initiatives driven by the provincial ERAS team. They work closely with unit leaders to improve management practices such as team meetings, visual displays of ERAS data and they facilitate PDSA (plan-do-study-act) cycles, and oversee implementation and spread of changes required to be “compliant” with best care. The use of the nurse coordinator role was an enabler of building quality capacity at the front lines and as a communication vehicle to ensure compliance and expectations from leadership were followed through on.

The experience of ERAS Implementation in Alberta Health Services highlights important themes, key messages and healthcare leader perspectives on what was required to move ERAS beyond implementation in one surgical area to spread and scale across the health system. The interview data provided sufficient breadth to illustrate a chronological order to characterize the evolving roles required of AHS leaders involved in ERAS to move from implementation to sustainability. They also illuminated the need for vertical Integration of Knowledge Translation linking the patient, provider, and system levels to address barriers and enablers to quality care across levels of a typical healthcare organization. The current research emphasizes infrastructure and system level supports required to move ERAS to everyday practice.

The work to date [5–7, 12] reinforces the role of a bottom-up approach targeting stakeholder engagement, ERAS as the standard approach, the role of education and training, the opportunity to involve patients [14], the importance of audit and feedback [22] visibility of the initiative and the role of nurse coordinators and champions. In order to drive health system transformation we can build upon drivers of High-performance learning systems that engage at the front lines combined with the role of leaders in top-down management strategies that focus on system metrics, process and outcomes.

Sustaining improvement in surgical care is essential. Interviewees (health system leaders) and key tasks have been mapped onto based on the IHI architecture of a high performance Management System [10]. Consistent features of high-performance health systems include a focus on explicitly organized front-line management with unit leaders guiding the day to day evidence based ERAS care by front-line staff. At higher levels of management, they document the role of an integrated management system architecture to enable quality planning and to reinforce and support work at the front line. In their recent review IHI describes key processes, structures and cultural norms as necessary factors for the establishment and maintenance of a high performance system at the front line, focusing on drivers related to Quality Control, Quality Improvement and Quality Planning. In AHS, Quality Control and Quality Improvement are linked and as the ERAS work matures and the health system evolves with adoption of a provincial electronic medical record and other quality approaches in surgery, Quality Planning can continue to support fundamental revisions in an aggregate and aligned fashion.

The ERAS implementation and its spread and scale across AHS has built a solid bottom-up and top-down foundation that reiterates the vertically integrated approach detailed by the IHI^10^. Within AHS we sought to inform system level strategies to enhance implementation, spread, scale and sustainability of ERAS approaches given that much of the evidence comes from patients, physicians and front line staff unit managers working at the patient care interface. The ERAS nurse coordinators as QI specialists, support managers at the level of the unit and the site (Tier 1 leaders). In the IHI work Tier 1 leaders are accountable for quality control and operations management, guiding the direct provision of care. Leaders at a higher level in AHS working across site, zone and provincial level need to initiate, guide and monitor the improvement at the system level. They are accountable for providing operational guidance and alignment with the overall organizational goals and strategy and they need to prioritize, tailor and respond to the QI opportunity with increased scope (IHI). These capabilities can be customized to any health system and of critical importance is the line of site from the top to front lines.

The findings of this paper identify that to create capacity for spread, scale and sustainability of ERAS at a health system level, unique approaches and strategies can build upon bottom-up and top-down strategies. Quality control and quality planning must be adopted and reinforced by healthcare leaders at all levels with a focus on ensuring a supportive environment, capacity, alignment and prioritization. Site based physician leaders, administrators and clinical leaders in nursing, pharmacy, nutrition, physiotherapy as well as provincial leaders working at the level of the health system play an integral role across key areas including data measurement and reporting, outcome assessment, change management and setting expectations to enable culture change through consistency of application and assessment of compliance to ERAS care. Leaders are in a unique position to effect and support a culture change in surgery through embracing standard work in a transparent system in which problems can be identified, understood and addressed by front line staff.

### Strengths and limitations

This work is intended to illuminate the role of leaders in sustainability of ERAS. Our interviews included a large number of champions and nurse coordinators and fewer numbers of leaders at the zone and provincial level. This may have skewed the results. In addition, although the TDF is the theoretical framework that we aimed to apply, the data was not captured or analyzed to give detailed results for approaches to change management for leaders such as through the behaviour change wheel [23]. Rather, it was analyzed to provide a high level overview for next steps in the organization in its goal of being a high performing healthcare organization.

## Conclusions

ERAS leaders have unique perspectives and approaches to support spread, scale and sustainment of ERAS that are different from those of site based ERAS teams. Leaders need to support sustainability of this work through a variety of methods including integration of QI goals, defining and communicating standard work, supporting front-line teams with implementation and integration. The ERAS nurse coordinators are implementation and quality specialists who are vital to the success of leaders at the unit level. Their work needs to be supported at the zone and province level through coordinated Quality Planning. This work informs us of gaps that need to be addressed in order to sustain and to continue to spread and scale ERAS strategies for all surgery patients.

## Supplementary information

**Additional file 1.** Question Guide

**Additional file 2.** Stages of ERAS development

## Data Availability

All data generated or analysed during this study are included in this published article and its supplementary information files.

## Abbreviations

ERAS: Enhanced Recovery After Surgery
AHS: Alberta Health Services
QUERI: Quality Enhancement Research initiative
TDF: Theoretic Domains Framework
SCN: Strategic Clinical Network
PRIHS: Partnership for research in the health system
