# Supplementary material for: Moving enhanced recovery after surgery from implementation to sustainability across a health system: a qualitative assessment of leadership perspectives
Source: BMC Health Serv Res. 2020 Apr 26;20:361. doi: 10.1186/s12913-020-05227-0 (PMC7183608; doi:10.1186/s12913-020-05227-0)
Supplement: Supplementary file 1 — Additional file 1. Question Guide [file 12913_2020_5227_MOESM1_ESM.docx]

Appendix 1: Question Guide

1. Unit of Analysis: What is your site, profession, role in ERAS implementation
2. What elements of the ERAS implementation were helpful in encouraging change? What has gone well for yourself, your team, your site
3. What could have been done differently? What were the gaps? What was difficult?
4. What impact has the implementation of multiple guidelines had at your site?
5. Have ERAS practices migrated beyond official ERAS surgery at your site?
6. What hospital, zone and AHS level issues and initiatives have impacted ERAS implementation at your site?
7. What are your recommendations going forward regarding ERAS implementation, spread, scale and sustainability?
