# Supplementary material for: Moving enhanced recovery after surgery from implementation to sustainability across a health system: a qualitative assessment of leadership perspectives
Source: BMC Health Serv Res. 2020 Apr 26;20:361. doi: 10.1186/s12913-020-05227-0 (PMC7183608; doi:10.1186/s12913-020-05227-0)
Supplement: Supplementary file 2 — Additional file 2. Stages of ERAS development [file 12913_2020_5227_MOESM2_ESM.docx]

|  | Stages of ERAS development | | |
| --- | --- | --- | --- |
|  | Initiate & Implement | Spread & Scale | Sustain |
| Themes |  |  |  |
| Supportive Environment |  |  |  |
| Leadership | *We had very strong physician leadership from the beginning, both with general surgery, the colorectal guys, as well as with anesthesia. The fact that they bought in right from the beginning and were visible champions, I think that made a huge difference in terms of being able to move ERAS forward. Once that commitment was seen I think we saw the nursing leadership, the operational leadership also make that commitment. And so, I think that’s really key in this work.*  Clinician_11 | *So my Executive Director is very, very pro-ERAS which is a big help as well as my unit manager, patient care manager for that inpatient unit. They’re very proud of ERAS because I am constantly told that ERAS started here at [site] and that they have been doing it the longest and that they are very, very pro-ERAS. And certainly at the sort of executive level there’s a lot of interest and desire to add additional services surgeries to ERAS. At least here, if we can get sustainment going for colorectal at some point that they’re certainly very supportive and would like to expand if possible at some point. I have found my site administration to be particularly helpful when there is some resistance from management or educators, that sort of thing having that sort of above everybody [Laughs], you know top down sort of. And I don’t want to say that, ‘yes I need her to be punitive towards other people’, it’s not that but because she’s for it she makes it clear to other that ERAS is a priority rather than…sort of you know you can do it if you feel like it sort of thing. it’s very, ‘nope this is what we do this is our standard of care here’.*  Clinician_10 | *The fact that all zones want to be involved in ERAS has been a good thing. I think the engagement at the site level has been really really positive, the site operating officers. The zone meeting- the zone leadership may be less engaged but from my perspective when we sold the spreads strategy, it was sold to the executive leadership of each zone. They are the ones that put their hands up and signed up. I’ve seen less of that than maybe some other people do, but I am not in the trench with it, so there may be a different dynamic that is going on at a zone level that’s different to what I’ve seen at a provincializing of funding, a translation evidence production perspective. Clearly, everyone at the C suite speaks of ERAS as a big success of bringing evidence into practice.*  Provincial_14 |
| Resources | *We have a dedicated manager for the ERAS program. So she organizes all of our team meetings, she invites people and she has a little bit more, I don’t know, power I guess. And so she can influence things like finance. She can make the managers of the units come to these meetings because she is their manager. So having her is actually very, very helpful. So I think that anybody that can get a site manager on their regular teams, should. And plus, she knows the ins and outs and stuff. Like, if we’re looking for a new product, she knows where to go.*  Clinician_1 | *From the system wide, running the program thing…I think that if we are going to implement this across the entire hospital, which is eventually where I think we need to go, you know vascular, urology, gynecology, or orthopedics. There is going to be some more financial support for nursing management, for the enhanced recovery after surgery coordinator nurse position. Not only for management program, but also for data analysis work that needs to happen. So you know more funding for that.*  Physician_4 | *You have to pick and choose where you’re gonna spend your resources because it’s not an open wallet to just keep hiring more people, more people, more people. So we have to kinda look at sustainability for ERAS so that multi service sites can now do some data collection on the new services that they have added, so I think that they’re having to make some choices. I’m not really sure how this is going to work, but [name 1] was telling me yesterday that we are probably gonna stop doing as much data collection as we have been doing cause it’s very labor intensive. The only thing is, if we’re not doing data collection, how do we know that we’re sustaining or improving and haven’t declined in any aspects if we’re not measuring it? So, that’s something I probably should talk a little bit with [name 2] about as to what that looks like down the road.*  Clinician_8 |
| Data | *When it comes to quality improvement, when it comes to trying to move forward change in the clinical environment data validation plays a major rule in getting teams to participate. Both the nurses on the floor, physicians to change practices we all know it can be difficult, probably just equally as difficult as other designations to get physicians to change their practice or to do something different from what they have been doing in the previous 5, 10 years. I felt the satiated approach with ERAS and the well documented 22 elements was a good contributor, being able to tap into the stories of EIAS data base and ERAS literature and data already collected were good tools for storytelling.*  Clinician_12 | *Yes, data collection at a multi guideline site is consuming. Very consuming. It seems like a constant juggle to try and just keep up. Because of course now you’ve got a site that’s saying “oh you know I think it would be valuable for us to have a newsletter, and we think it would be valuable if I have four post op units that we have new whiteboards every month” and you think wow we are talking about significant amounts of data time and taking away from unit time, in lack of better words. Yeah data collection is consuming. Often I feel like I am collecting data but I don’t get a chance to really look at it, regurge, and share it and just kind of putting pieces together and I’m thinking “okay this should work” instead of making it meaningful and more working with the staff to engage them on “hey so what do you think about this red zone”….And that is something I forgot to mention too is multi guideline, the benefit is scale and spread, that people want in. They are excited about it. They are excited to see what you have to say…. Again it’s the data. The proven results not only captures- it actually captures less attention to nursing and more to physicians. But nursing are interested in all the little pieces of “how did you get here?”, “what has your shift been thinking?”, “how did you get here?”, “What did you do?” It’s not just one piece it is many pieces.*  Clinician_2 | *So, it’s kinda tricky that way. I would like to see it expand to all the surgeries and have ERAS for all because it does make a difference. I’m worried about sustainability because ERAS for colorectal has been at this site for quite some time. But research has shown that if the data isn’t monitored, it does drop off and it’s so true. They’re just not self-sustained. They need somebody to still have the meetings, still bring the group together, still talk about the data otherwise I’m fearful that it will just drop off.*  Clinician_15 |
| Coordinators & Champions |  |  |  |
| Coordinators | *From what is working, I mean definitely having a nurse coordinator at each site, that’s working. And I see that is very important, especially up front to getting it launched, having the connections on site…. So having the onsite at the beginning of the implementation is definitely working*  Provincial_08 | *I mean there’s a big culture shift that needs to happen…. But it’s a shift that I think we need to start working towards and just staying on the top. Like orthopedics, they’d be a great target group, very low cost and probably get some good benefit out of them. We need to have the resources, so like the ERAS nurse clinician level to allow that to happen. ‘Cause I’m not sure that she’s got all the support that she would need to roll this out beyond colorectal and continue to follow it. I will say that, because she is so overloaded with multiple aspects of ERAS, whereas I’m just focused on the intraoperative part primarily, there’s just not enough of her to go around to also educating and helping implement in a due area. So I would say that that’s right limiting staff is that there’s just simply not enough hands at that level, at the nurse clinician level to proceed things forward and roll them out beyond our colorectal patients.*  Physician_7 | *I am seeing this project is doing great, but I do hear when I meet my team, I do hear that though we talk of sustainment, everybody ultimately is worried about- because a lot of our team members are temporary, and so when we talk of sustainment, we need to have that continuity and I hear from them that ‘What happens after the position ends?’ ‘What if that person doesn’t continue?’ I think that is a very valid question because if we have a new person, and we have this person with a lot of experience who has built over one year we are losing that person and then we are trying to create everything new from scratch. So that could be one thing but overall looking at the project, the work, I think it’s doing great and there is a lot of room for improvement, like I said documentation and there is lots to go, because we are finding a lot of gaps.*  Provincial_11 |

| Champions | *It’s absolutely vital that there’s champion and both formal leadership champions and informal champions and leaders for each of the professional groups, for each of the clinical service areas... And every place along the pathway that there’s patient care delivered, patient service delivered, there has to be champions around making things better for patients and providers. And if the focus is not on both of those, then it doesn’t work.*  Provincial_13 | *Certainly for the newer groups, they’re just trying to get used to change in practice, change in processes as well too, which could explain why the compliances are not so high at first. Each site has its own characteristics as well, their own barriers as well as enablers as well. Whether it be, do they have the type of champions that they have, it could be that as well. And what model is working for that team to move forward. So I think, we could certainly provincially provide guidelines but it’s the sites that will take a look and see what will work them. It’s not a cookie-cutter sort of thing. Right?*  Provincial_02 | *Even when they roll out these types of things, even if we have the resources in place to roll them out, we’re not great at considering the impact of rolling out multiple pathways in light of whatever else is going on and the people who really feel that are the administrators. The docs don’t really feel it, even the nurses don’t sometimes feel it, and it’s the administrators who really feel it. They’ve got one administrator for multiple sites, for multiple portfolios, who all of a sudden have a bunch of people who they are expected to reach out to and get them to champion and they are expected to promote not just one pathway but five or six pathways.*  Provincial_04 |
| --- | --- | --- | --- |

| Building Capacity |  |  |  |
| --- | --- | --- | --- |
| Change Mgmt | *So, I was just identified as one of those people that could help spreading that culture change. And I think that people need a variety of formats, they need multiple, multiple, multiple opportunities to see it, to walk past it, to hear about it, to be forced to listen to it [chuckles], to be not forced to listen to it. It just has to be around. And then you have to just promote best practice and try to speak to their, hopefully, inherit desire to do good. I think some people want research, I think some people want praise, I think some people want to be followers, some people want to be leaders, and I think you have to have a little bit of something for everyone to do a culture change.*  Clinician_5 | *In terms of challenges, the biggest thing was just the constant need for reinforcement about what we were doing, both on the surgeon side and the nursing side. That takes, once you implement a change…it seems to take a couple of years for them to adopt it and use it on a daily routine basis. Just ‘cause you’re so used to doing things the old ways. So I think the challenge is that people tend to fall backwards and the solution to that is just constantly reinforcing the value…*  Physician_6 | *P: To keep this focus that there’s practice changes at an individual clinician level and then there’s practice changes in all the people around that clinician. So if it’s a physician or a nurse, there’s day to day practice changes and then all the pieces around that person who’s touching the patient have to also change, which can be everything from where is the oral nutrition supplement actually kept on the unit and then what’s the documentation and the triggers to actually have that happen.*  *It’s a massive system level changes, even at the individual clinician level to support that individual. And so there’s been a whole variety of activities at the sites from staff education to changing our forms to all the different logistics on a unit or on a clinic to have the practice with patients change.*  Provincial_13 |
| Education | *In actual fact we had started some of the ERAS principles long before we started the process. We started the formal ERAS and because we been aware of it and informed and been to meetings about them. I think the education part of that so the roll out was the real key in my perspective. I don’t really know of anything else that I would have changed or done differently.*  Physician_13 | *The thinking about mobilization and nutritional supplements and the modern fasting guidelines, we are not just initiating that education and teaching with the colorectal patients. We talk about that with all of our patients that are coming through the pre-admission clinic for all of our services. So, I think that there are a lot of aspects of ERAS that have sorta migrated over. The mobilization, the intraoperative fluid, I think we’re seeing more and more of that just become the change in how we deliver care. So I don’t think it’s a big leap to go add another service on full bore. That will be really easy because, like the nursing personnel, ERAS way of managing patients is now become the way they do care….* *I think most units and areas embrace this because it is better for the patient.*  Clinician_8 | *There had already been some learnings done so they knew barriers and they were able to remove it…but the way we are going, it’s going to take forever… why wouldn’t we just lift the whole thing up? So we use one or two as a pilot and now let’s roll them all. If they have ERAS international guidelines… So we started with colorectal surgeries, then we went to women’s, but you are constantly just working on one small area. Why wouldn’t you just bring some resources to a site and do all the surgical programs? So then you have all the education. So then your floats, it doesn’t matter where they go or your educators, it doesn’t matter what unit you are on, or it doesn’t matter for nurses if I get an off service patient…So I agree that you need to start with one or two pilots and kind of work with the site to identify barriers and anything like that and start that kind of stuff but then it’s almost better just to lift the whole program, all surgical subspecialties. Put enough resources in to support them all.*  Clinician_13 |
| Teams | *I think we worked as a team. We had regular meetings for feedback. And if the ERAS nurse had a question and we usually addressed it quickly. At least we tried to. We would brainstorm for things that hadn’t happened yet… Sometimes different physicians they are used to doing certain things in a certain way so they might not implement as early as others. People who kind of … people who jumped on the ERAS band wagon. Some people are more resistant to change than others. But overall it was slowly but surely… Everyone helped each other. Everyone has their own silo but overall people have been helpful. But there is an ERAS nurse that helps to put it all together.*  Physician_12 | *The learning collaborative were really effective way to bring teams together and have that conversation. And as you spread to so many different teams, the capacity to do those same kind of learning collaborative. So I don’t know how the teams currently are really actively working towards some of the changes and when you have less coordinator time as well I suspect as just with the spread and capacity there are some challenges in to moving the dial.*  Provincial_06 | *Absolutely! You know we have multiple examples of that in our colorectal data. There is no doubt about it that they reciprocated back to what was comfortable, or what they sort of used to do, or what is the normal routine. I think there are a number of things that we are trying to do. Number 1 is show the successes. I think it is really important to show the successes to not only the ERAS group, like the actual ERAS people that are leading this but the group as a whole, the actual people that are doing the work. And not just the surgeons, not just anesthesia, but the nurses and all of the people who are involved in, the ward nurses the PHU nurses, the OR nurses, all of these people like the day surgery, the pre-op clinic people. All of these people need to see these successes and they show that this makes a difference, this is saving money, this is improving patients outcomes, this is decreasing length of stay, show that they decrease their urinary tract infection rate, decrease their whatever complication rate you want to choose. So I think that that cannot be overstated enough, I really believe that to involve and engaging that group as a whole not just the physicians not just a specific portion of the group, but the whole team as a whole and all of the cogs in it, because I think that’s how you get buy-in.*  Physician_2 |

| Barriers & Enablers |  |  |  |
| --- | --- | --- | --- |
| Outcomes | *So I think that showing people the data that good practice leads to good outcomes was a very powerful tool in- that’s probably the most convincing thing I could do…I guess it would be a short term laboring, that’s a management problem - where you do the change and you measure outcomes you get a short term view. So I think that was very good for that. I think that was the most powerful tool that I had. Again, the coordinator was very supportive of that.*  Physician_8 | *P: I think to encourage the surgeons and get them more involved, allowing the surgeons at various sites to or make it more easily accessible to access the database to do research would be one real key. I also think for a while we were tracking individual surgeon outcomes, which is something that gets done in NSQIP only to give them to individual surgeons for feedback on their individual compliance. And apparently the advisory panel nixed so we had to scramble in that, but having individual surgeon results that are kept confidential but pass back to surgeon involved to show their compliance, show their outcomes is a great stimulus for compliance. Because most surgeons are fairly type triple A competitive, we all want good results, the only way you know if you have good results if you see your individual results. So seeing results from the whole site is one thing, seeing your individual results and how you compare to other surgeons at your site as opposed to the whole site in a confidential manner, I actually think that’s very useful and is a stimulus to make people want to comply.*  Physician_13 | *What might help with that tension, is to focus on outcomes and not on compliance. The outcomes for patients’ providers and the system are the why we need to do this. And looking at what needs to change, how do we actually know that it’s changed? We need to look at that with a whole bunch of different lenses…. The gains, especially related to length of stay, readmission, is extremely powerful and we need to focus on that. We need to focus on the patient outcome data and the other piece that we need to find some ways that are not labor intensive to tap into the data that’s actually already available related to patient satisfaction and then do some creative pieces on patient experience*  Provincial_13 |
| Multiple Guidelines | *So I think we are just kind of getting into a discussion at [site] in terms of where do we go from here? So for sure we need to sustain the work that’s been done with colorectal but then how do we look at spreading it and what makes the most sense…. Do you pick one element and spread it across multiple services or take the next service and fully implement. So I’m not sure what the best answer is there, but how do we move forward in a way that then integrates it into practice and makes it become sustainable I guess.I would actually be very interested in hearing some of the experiences of the folks that have implemented across multiple services at their sites, because I feel like I can actually learn from that experience to help us determine where we go from here.*  Clinician_10 | *As we are moving along as a multi-guideline site, some of the you know spread and scale of ERAS and lots of movement and activity with ERAS, the ability to support the other CNE’s who are going through it ‘cause you are not just- it’s very challenging- you’re not just touch pointing one unit you are actually on several spaces. So, it’s all the intake areas its surgical areas, recovery rooms, and there are so many areas. For the [site] there are 10 different areas we must consider, so that is a significant amount of education to provide.*  Clinician_2 | *[Implementing one pathway…] it seemed to be very doable. When we got to multiple pathways, it wasn’t that it wasn’t doable, but what I started to hear from the sites…was more of a little bit of a sense of exhaustion and frustration with multiple things coming at once. So I started to hear “Okay I can handle one pathway” and especially from the site sponsors right? Through the site administrators, they are kind of like “yeah I can tap my one physician on the shoulder” around this one pathway. But when there is multiple pathways I’ve started to get calls from them or hear from them more about general frustration with provincial teams, with the networks. And I think it’s not because they don’t know it was coming but because it felt, all the sudden it felt a little bit overwhelming. Because we were recruiting people at the same time and then we were rolling out and that requires a larger role for some of the administrators.*  Provincial_04 |
| Consistency | *The way it was implemented, the foundational pieces weren’t put into place up front, so something as simple as templates, you know what they use for communicating to the sites, even posters, newsletters, when they are using it at the site. You know everyone had to use baseline data, but there wasn’t really a set template for that. So all those like foundational pieces, everybody knew that they had to do it, it’s just kind of was done, but it wasn’t documented in a uniform way…. I think the program would have been more, would be more successful if the foundational pieces would have been- if some time was spent up front putting this into place before they launched- the launching and then trying to document.*  Provincial_08 | *ERAS should become best practice for all patients. We need these standardized order sets for all patients - have to be out of bed day 0, all patients should have Ensure, etcetera. Then there would be a better uptake. But because we only have say 25-50% of patients are ERAS on units, I am speaking to post op, they’re the patients that are the most work. When really they are the patients that are getting the best care, if these measures are being done…. I don’t like to say ERAS anymore as I say enhanced recovery, because I feel like ERAS has gotten this negative definition to it where when you say enhanced recovery you can’t argue with that, right?*  Clinician_16 | *I think is also important especially with the consistency in provincial, in having, not the sites working in silos so even when we did the definition that was collected, or supposed to be collecting it this way every single time, so that’s been a change. But I have seen other opportunities where sites have talking more, they’re collecting more, they’re meeting more around either data collection or guidelines, or implementing new launching control. I think that’s also really important that one site cannot just be its own site…. I think breaking down some of those barriers started to happen when you started to roll out new provincial resources and information.*  Provincial_09 |
| Compliance | *So to see why isn’t there a compliance in this particular area I think – and I know it’s being done already – you need to drill down to the site; what’s happening at the site, what supports do they need, what’s really going well for them and what are some things that they feel they need some support on? And they’re the experts on the unit, on what’s working, what’s not working. I think to have the compliance become more successful in that particular area, it really has to come from the unit or the site. It’s something that will work for them.*  Provincial_02 | *I would like to see full scale implementation, I would like to see spread across the sites, and I would like to see our compliance figures go up. I realize that compliance the way they define it is the wrong way to define it but I’d like a subset of compliance that we buy into and then monitor that subset. So that we can truly say that people are getting the ERAS way.*  Provincial_14 | *Some it is organic, because everyone is getting off ward with some of the basic elements of ERAS. Some of the fine tuning stuff that are harder, like the big changes I think have been done, but some of the fine tuning in improving your compliance, no really active process on that. It’s challenging and truthfully I don’t how all of that is being achieved right now without having the learning collaborative and some of the provincial dedicated support really hands on. I think that the teams are doing as well as they can but I am sure it’s a challenge with the capacity and the ability of the coordinators to really be as involved because what we see is, unfortunately, without really good dedicated coordinator time hands-on on the unit working with the nurses, working with the physicians, advocating for change constantly, it’s really hard.*  Provincial_06 |
